# Expression of Concern: Masataka, N. Possible Anxiolytic Effects of Cannabidiol (CBD) Administration on Feline Responses to a Fear Response Test. *Animals* **2025**, *15*, 1642

**DOI:** 10.3390/ani15162365

**Published:** 2025-08-12

**Authors:** Animals Editorial Office

**Affiliations:** MDPI, St. Alban-Anlage 66, 4052 Basel, Switzerland; animals@mdpi.com

With this notice, the *Animals* Editorial Office alerts the readers of concerns related to this article [1]. Following publication, concerns were raised to the Editorial Office regarding the degree to which this study **adheres to MDPI’s policies on Research Involving the Use of Animals** (https://www.mdpi.com/ethics#_bookmark10 accessed on 23 July 2025). In order to maintain the highest scientific standards, the Editorial Office has decided to issue this expression of concern while an investigation is ongoing.

The author of this publication has been contacted for further clarification. The investigation is being coordinated by the Editorial Office, with the involvement of the Editor-in-Chief. Once this process is complete, the Editorial Office will provide an update on this situation and announce any post-publication modification deemed to be necessary, as per MDPI’s Updating Published Papers policy (https://www.mdpi.com/ethics#_bookmark30).
